# Editorial: Novel and Improved Methods for the Prevention and Treatment of Graft-Versus-Host Disease (GVHD)

**DOI:** 10.3389/fimmu.2022.966389

**Published:** 2022-06-30

**Authors:** Evelyn Ullrich, Andreas Beilhack, Dietlinde Wolf

**Affiliations:** ^1^ Experimental Immunology, Children’s University Hospital, Goethe University Frankfurt, Frankfurt am Main, Germany; ^2^ Frankfurt Cancer Institute (FCI), Goethe University Frankfurt, Frankfurt am Main, Germany; ^3^ German Cancer Consortium (DKTK), Partner Site Frankfurt/Mainz, Frankfurt am Main, Germany; ^4^ Department of Medicine II, Center for Interdisciplinary Clinical Research (IZKF), University Hospital Wuerzburg, Wuerzburg, Germany; ^5^ Sylvester Comprehensive Cancer Center; University of Miami, Miller School of Medicine, Miami, FL, United States

**Keywords:** allogeneic hematopoietic stem cell transplantation (aHSCT), acute and chronic graft versus host disease (a/c GVHD), GVL, metabolism, microbiome, cell therapy

For patients with a variety of severe diseases, including primarily hematopoietic malignancies, immunodeficiency syndromes, and genetic disorders, allogeneic hematopoietic stem cell transplantation (aHSCT) represents a potentially curative therapeutic approach. In this context, despite significant progress in the optimization of aHSCT, the development of graft-versus-host disease (GVHD) remains a challenge to long-term transplant success after aHSCT. It is associated with significant morbidity and mortality and is the major cause of non-relapse mortality.

GVHD occurs when donor T cells are primed by recipient antigens subsequently eliciting an inflammatory response against the host. Clinically, two types of GVHD are distinguishable: an acute form (aGVHD), and a chronic form (cGVHD). In brief, the main characteristics: aGVHD occurs in 30-50% of aHSCTs and is a multi-organ disorder resulting from inflammatory cytokines and donor T cells which primarily damage skin, liver, gastrointestinal tract, and eye. cGVHD, with a prevalence of 30-70% of aHSCTs, is induced by T and B cells resulting in a heterogeneous immunological complication affecting virtually every organ.

Traditionally, broad immune-suppressive drugs (with considerable toxicities) including calcineurin inhibitors (CNI) (cyclosporin or tacrolimus), together with methotrexate or mycophenolate mofetil (MMF), and mTOR inhibitors (Sirolimus/Rapamycin) are used as GVHD prophylaxis. But despite first success reports, significant GVHD still occurs with these drugs. Other prophylaxis strategies like pre-transplant anti-thymocyte globulin (ATG) are effective in reducing severe GVHD but have no survival benefits and steroids have serious side effects.

One of the most critical challenges in aHSCT is the development of less toxic and more targeted therapies that maintain the graft-versus-leukemia/tumor (GVL/T) effect but suppress GVHD while facilitating enhanced immune reconstitution relative to existing strategies. Recently, several prophylaxis strategies for GVHD have been developed and others are currently in development, including, for example in the case of haploidentical HSCT, post-transplant cyclophosphamide (PTCy), which seem to be very promising.

In the frame of this specific Research Topic, we aimed to collect recent developments of innovative methods for both prevention and treatment of GVHD, without impairment of GVL. In 8 original research articles and 4 reviews, this edition provides a deep insight into the role of the microbiome and metabolism as well as recent advances of small molecule and cell therapy development.

We are glad, that experts in the field highlight the recent progress in the broad field of immune cell metabolism with two comprehensive reviews. Mohamed et al., summarize metabolic pathways contributing to GVHD and discuss metabolic targets for acute and chronic GVHD in immune and non-immune cell as well as the immunomodulatory function of microbial metabolites. Furthermore, they examine the metabolic effects of co-inhibitory pathway blockade (PD-1) and cellular therapies (Tregs/MSCs/Bregs) in aHSCT. The mini review by Karl et al., provides an overview of metabolic T cell alterations in GVHD and illustrates the impact of conventional GVHD therapy on T cell metabolism.

Recent studies have shown the association of microbiome dysbiosis and aGVHD. Here, primary research by Ghimire et al., investigates the role of G-protein coupled receptors (GPR43 and GPR109A) which engage microbial derived metabolites, like short chain fatty acids, in the mitigation of GVHD in intestinal biopsies from patients after allo-HSCT. A second study by Heidrich et al., describes an association of dental biofilm microbiota dysbiosis with the risk of aGVHD.

In the context of cell therapy development, the biological relevance of T helper cell lineage defining transcription factors as potential targets for GVHD therapy has been delineated in a review article by Campe and Ullrich. Moreover, Agbogan et al., explore the immunomodulatory effect of adoptively transferred CpG-activated B cell progenitors to alleviate GVHD symptoms. In addition, Scheurer et al., describe an *in vitro* generated sub-population of CD11b+CD11c+ myeloid-derived suppressor cells (MDSCs) as potent immune modulators leading to the prevention of GVHD without negatively affecting tumor cytotoxicity. Another innovative and attractive strategy using CRISPR/Cas9 has been described by Majumder et al., for genetical engineering of naïve T cells pre transplant as a method for GVHD prevention in a major murine mismatch model.

In addition, the recent therapeutic advances in the area of drug development, e.g. small molecules and antibodies, are also addressed. Braun and Zeiser thoroughly review the role of kinase inhibition as novel treatment strategies for acute and chronic GvHD after allo-HCT. Thangavelu et al., evaluate the efficacy of a novel agonist of the retinoic X receptor (RXR), IRX4204, to treat cGVHD in two complementary murine models with bronchiolitis obliterans or sclerodermatous manifestations. Primary research by Matos et al., analyzes a possible association of anti-thymocyte globulin (ATG) treatment and serum levels of 25-hydroxyvitamin D3 and 1,25-dihydroxyvitamin D3 in 4 HSCT cohorts with different vitamin D3 supplementation. Lastly, Hadjis et al., characterize post-transplant cyclophosphamide as superior in ameliorating pre-clinical GVHD compared to five other optimally dosed chemotherapeutics (methotrexate, bendamustine, paclitaxel, vincristine, and cytarabine) that vary in mechanisms of action and drug resistance.

Finally, this Research Topic makes us again aware of how complex the regulation of GVDH is and in which fragile balance between GVHD and GVL patients after aHSCT find themselves. We are aware that this issue can only compile a first selection of innovative findings and treatment strategies that are currently being developed for the prevention and treatment of GVHD.

GVHD biology and treatment remains a field that is always influenced by current research developments and new advances can be expected in a short time. Therefore, we will continue to monitor the field and provide updates to the authorship of Frontiers in Immunology.

## Author Contributions

All authors listed have made a substantial, direct, and intellectual contribution to the work and approved it for publication.

## Conflict of Interest

EU is a member of Phialogics and receives research support from BMS and Gilead. AB is a scientific cofounder of Aamuthera Biotech GmBH and Dualyx NV.

The remaining author declares that the research was conducted in the absence of any commercial or financial relationships that could be construed as a potential conflict of interest.

## Publisher’s Note

All claims expressed in this article are solely those of the authors and do not necessarily represent those of their affiliated organizations, or those of the publisher, the editors and the reviewers. Any product that may be evaluated in this article, or claim that may be made by its manufacturer, is not guaranteed or endorsed by the publisher.

